# Working with goals in youth mental health and substance use service: A review of current practice guidelines and recommendations

**DOI:** 10.1002/jcv2.70173

**Published:** 2026-10-07

**Authors:** Madison Nugent, Drew Burchell, Jill Chorney, Sharon Clark, Mick Cooper, Debbie Emberly, Danielle Impey, Jenna Jacob, Anam Khan, Jennifer Lane, Duncan Law, Rebecca Pillai Riddell, Lori Wozney

**Affiliations:** ^1^ IWK Health Halifax Nova Scotia Canada; ^2^ Dalhousie University Halifax Nova Scotia Canada; ^3^ Office of Addictions and Mental Health Provincial Government of Nova Scotia Halifax Nova Scotia Canada; ^4^ School of Psychology University of Roehampton London UK; ^5^ Knowledge Institute on Child and Youth Mental Health and Addictions Ottawa Ontario Canada; ^6^ Anna Freud Centre London UK; ^7^ University College London London UK; ^8^ MindMonkey Associates London UK; ^9^ York University Toronto Ontario Canada

**Keywords:** goal‐oriented practice, goals in therapy, goal setting, measurement‐based care, mental health, psychotherapy, routine outcome monitoring, youth

## Abstract

**Background:**

Working with goals is a critical yet often overlooked aspect of youth mental health and addictions care, with the potential to strengthen therapeutic relationships, support shared decision‐making, and improve outcomes. Despite its importance, there is no cohesive, equity‐centered guidance for working with youth on their goals. This review critically examines existing practice principles for working with youth aged 10–25 on goals within mental health and addictions services, with a particular focus on how equity, diversity, and inclusion are, or are not, embedded in these recommended approaches.

**Methods:**

A narrative review methodology was employed to synthesize insights from both academic literature and clinical guidebooks published between 2003 and 2023. Searches were conducted across PubMed, EMBASE, Cochrane Library, Web of Science, and supplemented with general web searches to capture diverse perspectives from research and practice. Fifty‐three publications were selected and analyzed using thematic and content analysis via MAXQDA software.

**Results:**

Four key themes were identified: (1) semi‐structured and strategic approaches to goal‐setting, including the use of routine outcome measures and progress monitoring; (2) youth‐ and family‐centered engagement strategies; (3) conceptual framing of goals within therapeutic processes; and (4) a nascent but growing emphasis on embedding EDI and cultural safety into goal‐oriented practices.

**Conclusion:**

Findings underscore the importance of moving beyond standardized, clinician‐driven approaches to more inclusive, collaborative, and youth‐informed goal practices.

## Key Points

**What's known?**

- Goals are often set but not actively used in therapy, reflecting limited practice‐ready guidance in existing research. This review synthesizes fragmented evidence to demonstrate how goals can be meaningfully integrated and revisited throughout therapy, rather than treated as a one‐time assessment task.

**What's new?**

- Research rarely explains how goal‐focused work supports engagement in everyday practice. By integrating research findings and practice recommendations, this review clarifies how working with goals can promote shared understanding and engagement, without becoming rigid or administratively driven.

**What's relevant?**

- The literature often under‐represents the realities of youth‐focused and diverse service settings. This review highlights practical considerations for adapting goal‐related practices to developmental, cultural, and system contexts commonly encountered in youth mental health and addictions care.

## INTRODUCTION

Adolescence and young adulthood are periods marked by rapid biological, psychological, and social transitions that shape identity, relationships, and future health trajectories (Blakemore, 2019). These developmental processes occur within broader social, cultural, and structural contexts that influence young people's experiences of mental health and substance use challenges, including opportunities and willingness to seek support, but also whose needs are recognized and validated, and who is supported in accessing services (Clark et al., 2018; Guinaudie et al., 2020; Watson et al., 2023). Continuing to advance new knowledge about the essential factors in psychotherapy for young people and support continuous improvement in clinical practice is important to realizing therapeutic outcomes (Cooper et al., 2025).

### Goal‐oriented practices in youth mental health and addiction settings

Within the context of youth mental health and addictions, working with goals is viewed as a foundational component of therapeutic interventions and a mechanism that can facilitate shared decision‐making, strengthen the therapeutic relationship, and improve clinical outcomes (e.g., Cooper & Law, 2018; Law & Bradley, 2014; Tryon et al., 2018; Wolpert et al., 2016). Goal‐oriented practices encompass a range of therapeutic activities that directly address or explore the young person's treatment objectives and expectations, including goal formulation and discussion of goal progress through tracking or monitoring (Cooper & Law, 2018).

Central to many evidence‐based treatments for youth, such as cognitive‐behavioral therapy and motivational interviewing is the collaborative identification and pursuit of personally meaningful goals (Epton et al., 2017). A recent large‐scale study of 792 children and adolescents receiving low‐intensity cognitive‐behavioural therapy ,CBT demonstrated that progress on self‐defined goals, especially those centered on emotional awareness and expression, was significantly associated with reductions in anxiety and depression, even when controlling for session count (Smith et al., 2023). Complementing this, our 2025 scoping review of 116 studies revealed wide variation in how goals are set, tracked, and measured in youth settings (Jacob et al., 2025). The use of goal‐based measures and approaches is expected to grow globally, particularly as Integrated Youth Service models are advancing more youth‐centered approaches to care (Chiodo et al., 2022, 2025; Henderson et al., 2023). Yet despite its ubiquity, there are no universally accepted quality standards guiding clinical practices around goals in youth mental health and addictions contexts (Jacob et al., 2023). This gap extends beyond procedural guidance to limited consideration of ways of working with goals that embed equity, diversity, and inclusivity despite increasing recognition that quality of services for young people require approaches that actively address systemic barriers and support person‐centered practices (Alderman et al., 2024; Henderson et al., 2022).

The lack of clear practice guidance raises a critical concern: without intentional, high‐quality clinical approaches, goal‐oriented work risks becoming formulaic and disconnected from youth realities. Clinicians need tools and structures that support dynamic, reflective goal practices ‐ ones that evolve with changing evidence, youth identities, and system constraints. To address this, articulating a set of flexible, equity‐informed practice principles can help guide the “how” of goal work in the future would complement condition‐specific clinical practice guidelines with actionable strategies across a range of treatment approaches.

This paper aims to synthesize the current state of recommended goal‐oriented practices to inform development and dissemination of a set of core practice principles that support effective, equitable, and developmentally attuned goal‐oriented mental health and addictions care.

#### Goal formulation

There is evidence that goals play a pivotal role in building a positive therapeutic alliance and improving treatment outcomes (Jacob et al., 2018; Law & Bradley, 2014; Martin & Feltham, 2020; Tryon et al., 2018), ensuring young people feel more included in their care (Abrines‐Jaume et al., 2016), and reducing non‐attendance rates (Cairns et al., 2019). Though typically set during the early stages of therapy, goals may also emerge later in the process, and the content, focus, or wording of previously established goals may need to be reviewed and updated as a young person's circumstances, priorities, and treatment needs evolve (BACP, 2022; Jacob et al., 2022). Formulating goals in therapy might also facilitate young people and caregiver engagement and satisfaction with care (Cook et al., 2025; Jacob et al., 2021; Werntz et al., 2023). Cairns et al. (2019) identified a significant positive association between goal formulation and the number of sessions attended, while Jacob et al. (2017) reported that parents or carers of children with established goals expressed greater satisfaction with care compared to those without.

While the evidence supporting goal formulation is robust, there remains a notable gap in distilling how these practices are operationalized. Some point to the use of the SMART framework (Lawlor & Hornyak, 2012), while others advocate for more nuanced models such as Cooper's HEALSS CRISP framework (Cooper, 2019). This variability not only affects the consistency of care but may limit the effectiveness for equity‐denied communities. Clear, adaptable and feasible practice principles that reflect both clinical evidence and the social shifts in what youth and families need from their care experiences are needed.

#### Measuring and monitoring goals

Working with goals is often viewed as an iterative and collaborative process (Feltham et al., 2018), in which both the young person and clinician engage in discussions about which goals to pursue and how to work toward them (Tryon et al., 2018). Goal monitoring—the routine tracking of progress—typically involves self‐reported ratings on a numerical scale (Mok et al., 2024). A systematic review by Lloyd et al. (2019) found that using explicit Goal‐Based Outcome (GBO) measures can help ensure that both the practitioner and the client remain focused on the client's individual needs. A recent review (2025) identified a wide range of goal‐focused measures being applied. Tools such as Goal Attainment Scaling and the GBO tool (Law, 2019) are frequently referenced as facilitating progress tracking and ongoing evaluation of goals (Jacob et al., 2017, 2022; Kaminer et al., 2018; Lee et al., 2022). These tools support the translation of broad therapeutic aims into shorter‐term, attainable steps to improve engagement and clarity for both clinicians and youth (Cooper & Law, 2018; Gabrielsen et al., 2012; Jacob et al., 2017; Lavik et al., 2018).

Despite their promise, the application of goal monitoring tools and measures as well as use of data collection software and visualization technologies varies widely across settings. To ensure goal monitoring is both meaningful and equitable, there is a clear need for well‐defined practice principles that guide not only the selection of tools and measures but also their use in culturally responsive and developmentally appropriate ways over the full episode of care.

### Equity, diversity and inclusivity as foundations of goal oriented practices

While goal formulation and monitoring are established therapeutic activities, there is limited evidence on how race, ethnicity, gender, sexual orientation, and other aspects of identity influence their effectiveness (Lane et al., 2025). Embedding equity, diversity, and inclusion (EDI) into goal‐oriented practices in youth mental health care is not only ethically imperative, but also mandated by national and international policy frameworks that call for culturally safe, person‐centered, and rights‐based approaches. Recent directives from health authorities, research funders, and governance bodies (e.g., Canada's Tri‐Agency EDI Action Plan (Government of Canada, 2022), UK's NHS England Equality, Diversity and Inclusion (EDI) Improvement Plan (NHS England, 2023)) emphasize the need to dismantle systemic barriers and ensure that care is responsive to the diverse identities, lived experiences, and social contexts of youth. Goal‐setting, when approached through an EDI lens, becomes a powerful mechanism for amplifying youth voice, fostering trust, and tailoring interventions. Without this intentional integration, goal‐oriented practices risk reinforcing dominant norms and excluding marginalized perspectives, undermining its therapeutic potential. Aligning goal practices with EDI principles ensures that youth co‐creators of meaningful pathways to wellness rather than just participants in services (Duncan et al., 2025; Guinaudie et al., 2020; Henderson et al., 2022).

More inclusive and affirming therapeutic spaces for youth can be created by challenging Western clinical pedagogies and praxis (Alderman et al., 2024). One such framework is Cultural Safety, which originated with Māori nurse leaders and midwives in New Zealand in the 1980s as a response to colonization and systemic health inequities (Papps & Ramsden, 1996; Williams, 1999). Formalized across multiple contexts as a standard for service delivery, cultural safety emphasizes practitioner self‐reflection, shared respect and learning, and actions that recognize and respond to the cultural, spiritual, social, and emotional identities of individuals who ultimately determine whether the care they receive is culturally safe (Cultural Safety and Te Ao Māori, 2024; Nursing Council of New Zealand, 2002; Williams, 1999). Nonetheless, it is unclear how cultural safety and other lenses have or have not informed guidance on working with goals in youth mental health an addiction settings.

This review aimed to (1) extract and synthesize guidelines and recommended approaches for working with goals with youth aged 10–25 in community mental health and addictions settings, and (2) elucidate equity, diversity, and inclusion‐related considerations within those recommended practices including their hypothesized impact on working with goals.

## METHODS

### Overview

A review of academic and clinical literature was conducted. Unlike systematic reviews, which address narrow questions, narrative reviews allow for broader interpretation and critique across diverse sources (Greenhalgh et al., 2018). Following guidance for reflexive and rigorous searches (Ferrari, 2015; Sukhera, 2022), we defined scope, rationale, and inclusion/exclusion criteria. To conceptualize findings, we drew on thematic and content analysis methods (Braun & Clarke, 2006; Kuckartz & Radiker, 2023), including: (1) data familiarization, (2) initial coding using broad domains, (3) iterative clustering and refinement, and (4) summary reporting and visual mapping. MAXQDA 2022 was used for data extraction and analysis.

### Search strategy and inclusion criteria

A comprehensive search strategy as part of a broader scoping review published previously (Jacob et al., 2025) was co‐developed by an international Special Interest Research Group related to goals, the GBOM research team, and was led by an evidence synthesis expert. Sources included peer‐reviewed and grey literature, and regional, national, and international guidelines or clinical protocols as well as literature reviews and dissertations related to working with goals in youth community mental health and addictions services. The search encompassed databases such as PubMed, EMBASE, Cochrane Library, Web of Science. Example search terms included combinations of keywords and subjects related to goals (e.g., goal setting, goal‐based or goal‐oriented practice, treatment goals), youth populations restricted to ages 10–25 (e.g., youth, adolescent, teen, young person), and community‐based or outpatient mental health and addictions service contexts (e.g., psychotherapy, counselling, mental health practice, outpatient services), adapted from the search strategy used in the parent scoping review (Jacob et al., 2025). A complementary grey literature search was completed using general web searches with free‐text terms based on inclusion criteria to identify additional relevant guidelines not indexed in databases. Searches were limited to English materials published between January 2003 and January 2023.

#### Inclusion criteria

Included source material need to provide guidance on clinician practices related to goal setting, working with goals, or goal‐monitoring; be specific to community mental health and addictions treatment settings, and focus primarily on youth aged 10–25. This range aligns with the World Health Organization's (WHO) definitions—adolescents (10–19) and youth (15–24) and Integrated Youth Services (IYS, 2023) models (12–25).

#### Exclusion criteria

Publications that did not primarily focus on the age group of interest; were not specific to community mental health settings (e.g., inpatient hospitals, K‐12 classroom‐based interventions); or lacked relevance to the practice of working with goals in mental health treatment (e.g., focused on general therapeutic approaches) were excluded from the review.

### Literature selection

Initially, 124 records were identified from databases and grey literature. Twenty records were removed before screening for duplication or other reasons. Titles and abstracts for 104 publications were manually double screened, including 85 empirical journal articles, 1 non‐empirical publication, 5 dissertations, 13 clinical guidebooks. Two team members independently reviewed 85 full texts, with discrepancies resolved through discussion. A total of 53 publications were included in the review, as summarized in Figure 1.

**FIGURE 1:**
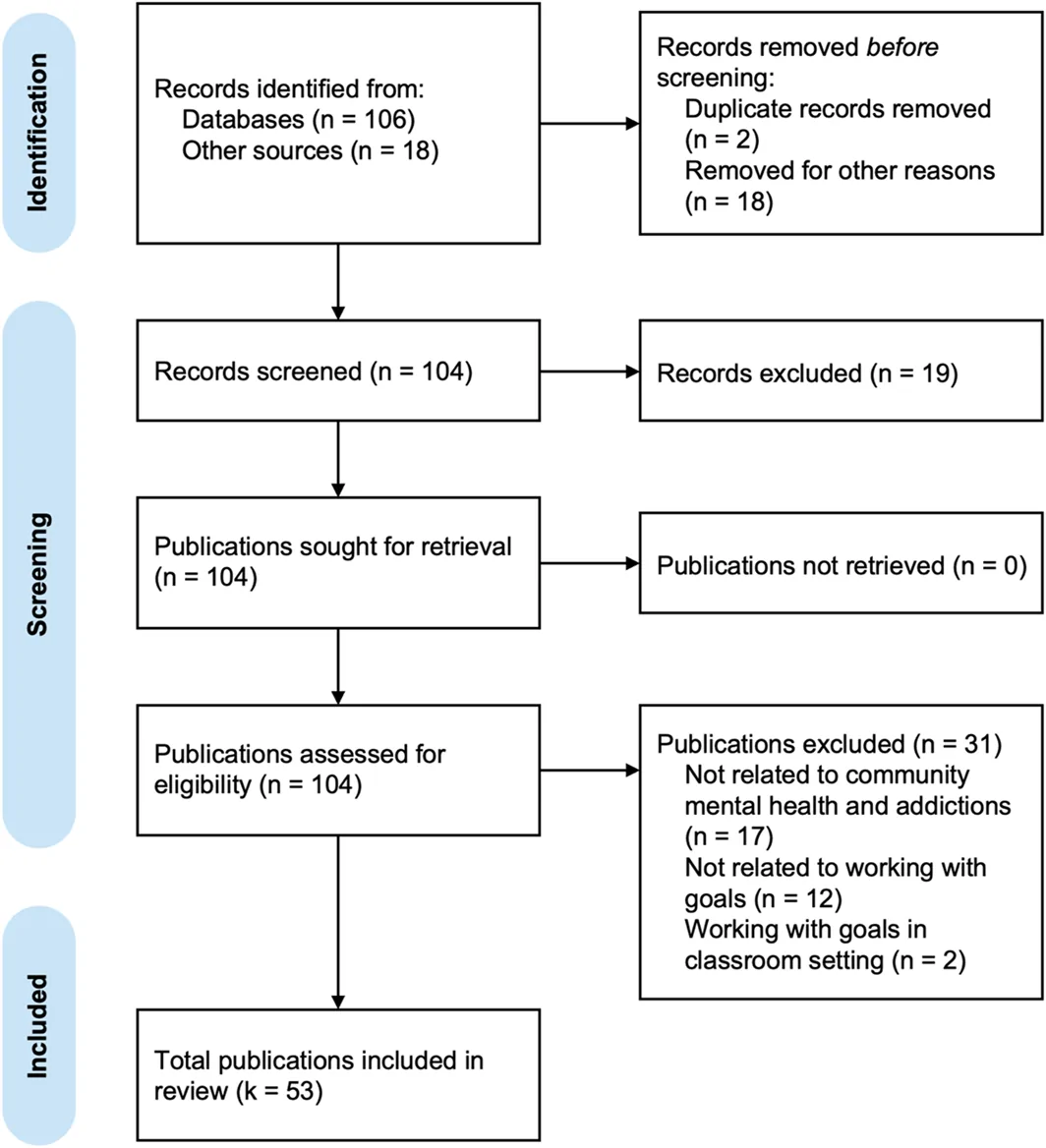
Flowchart of the literature selection process. *n*, frequency; *k*, total sample size.

Full texts were uploaded into MAXQDA. The highlighting tool was used to identify explicit or implied practice recommendations related to working with goals (e.g., statements advising or describing how clinicians set, review, monitor, or adapt goals with youth). Extracted segments included text that described recommended clinician behaviours, strategies, or structures for working with goals, rather than general descriptions of goals or summaries of results. Additionally, descriptive data were extracted (e.g., year of publication, country, treatment setting, population characteristics, etc.), as shown in Table 1. Regular meetings were held to iteratively develop the coding framework, refine definitions, and discuss ambiguities in coding informed by the hierarchical Code System feature of MAXQDA.

**TABLE 1:** Characteristics of included publications.

Publication	Sample size	Country	Type of publication	Population	Presenting problems	Therapeutic approach	Type of provider
Alderson et al. (2019)	<100	United Kingdom	Empirical, journal article, qualitative	Youth, parents, caregivers, clinicians	Substance use	Motivational enhancement therapy, social behaviour and network therapy	Not reported
Bhattacharya et al. (2013)	N/A	India	Empirical, journal article, narrative	Youth	General mental wellness	Intentional self‐development	Psychologist
Bhattacharya (2021)	<100	United States	Empirical, dissertation, mixed‐methods	Youth, clinicians	Multiple dx (depression, anxiety, general mental wellness, other)	Behavioural activation, positive psychology, CBT	Counsellor, psychologist
Bögels et al. (2008)	<100	Netherlands	Empirical, journal article, quantitative	Children, youth, parents, caregivers	Multiple dx (ADHD, OCD, autism)	Mindfulness therapy, mindfulness training for parents	Psychologist
Burton and Lent (2016)	N/A	United States	Empirical, journal article, narrative	Not reported	General mental wellness	SFBT, vision boards	Counsellor, psychologist
Cairns et al. (2015)	<100	Australia	Empirical, journal article, qualitative	Youth	Multiple dx (general mental wellness, depression, anxiety, PTSD)	Not reported	Not reported
Cairns et al. (2019)	101–500	Australia	Empirical, journal article, quantitative	Youth	Multiple dx (general mental wellness, substance use, other)	Not reported	Not reported
Carroll et al. (2013)	<100	Australia	Empirical, journal article, mixed methods (incl. case study)	Youth	Multiple dx (general mental wellness, substance use, other)	Self‐regulatory intervention, CBT, strengths‐based strategies	Counsellor, social worker
Chandrasekhar et al. (2023)	101–500	United States	Empirical, journal article, quantitative	Youth	General mental wellness	Psychoeducation program	Not reported
Choi et al. (2022)	101–500	United States	Empirical, journal article, quantitative	Children, youth, parents, caregivers	Autism	ABA	Not reported
Claessens et al. (2012)	501+	Uganda	Empirical, journal article, quantitative	Children, parents	General mental wellness	Psychosocial life‐skills intervention	Social worker
Cook‐Cottone et al. (2019)	N/A	United States	Empirical, book	Children, youth, clinicians	Multiple dx	DBT, CBT	Counsellor, psychologist
Cooper and Law (2018)	N/A	United Kingdom	Empirical, book	Not reported	General mental wellness	Not reported	Counsellor, psychologist, other mental health service providers
Cox et al. (2010)	101–500	United States	Empirical, journal article, quantitative	Youth, parents, caregivers, family members	Multiple dx	Strengths‐based approach	Counsellor, psychologist, social worker, other care team members
Day et al. (2017)	N/A	United Kingdom	Empirical, journal article, mixed methods (RCT)	Children, parents, caregivers	General mental wellness (parent with significant personality difficulties)	Parent education and training	Not reported
Dickens (2020)	<100	United States	Empirical, book	Youth, parents, caregivers, family members	Multiple dx (depression, eating disorder)	Family therapy, narrative therapy	Counsellor
Duong et al. (2016)	<100	United States	Empirical, journal article, qualitative	Youth	General mental wellness	Not reported	Counsellor, psychologist
Edbrooke‐Childs et al. (2015)	101–500	United Kingdom	Empirical, journal article, quantitative	Children	Multiple dx (emotional disturbance, eating disorder, self‐harm, conduct disorder, learning difficulties, habit disorder, et al.)	Not reported	Not reported
Fiks et al. (2013)	101–500	United States	Empirical, journal article, quantitative	Children, parents, caregivers, clinicians	ADHD	Shared decision making, behaviour modification interventions, medication	Counsellor
Francis et al. (2021)	N/A	United States	Empirical, journal article, narrative	Youth	Co‐occurring disabilities and mental health dx	Not reported	Counsellor, educators, other mental health service providers
Gabrielsen et al. (2012)	101–500	Norway	Empirical, journal article, quantitative	Youth	General mental wellness	Not reported	Not reported
Hanson and Lynch (2013)	N/A	United States	Empirical, book	Children, youth, parents, caregivers, family members	General mental wellness	Family‐centered approach	Psychologist
Hurlburt et al. (2010)	<100	United States	Empirical, journal article, quantitative	Children, youth, parents, caregivers, family members	Multiple dx (general mental wellness, ADHD, OCD, behavioural diagnosis, depression)	Child and family therapy	Counsellor, psychologist, social worker
Jacob et al. (2017)	101–500	United Kingdom	Empirical, journal article, quantitative	Children, youth	Multiple dx	Not reported	Not reported
Jacob et al. (2022)	N/A	United Kingdom	Empirical, journal article, narrative	Not reported	Multiple dx	CBT	Counsellor, psychologist, physician, occupational therapist
Jacob et al. (2023)	501+	United Kingdom	Empirical, journal article, quantitative	Youth	Multiple dx	Not reported	Counsellor, psychologist, psychiatrist
King (2009)	N/A	Canada	Empirical, journal article, narrative	Children, youth, parents, caregivers, clinicians	Multiple dx	Relational goal‐oriented model, relationship‐based practice	Counsellor, psychologist
Kaminer et al. (2018)	101–500	United States	Empirical, journal article, quantitative	Youth	Substance use	CBT	Counsellor, psychologist
Kanter et al. (2021)	101–500	United States	Empirical, journal article, quantitative	Youth	General mental wellness	Psychoeducation program	Not reported
Kiyimba et al. (2018)	<100	United Kingdom	Empirical, journal article, qualitative (incl. case study)	Children, youth, parents, caregivers, family members, clinicians, healthcare professionals	Multiple dx	Not reported	Not reported
Lavik et al. (2018)	<100	Norway	Empirical, journal article, qualitative	Youth	Multiple dx (anxiety, depression, trauma, self‐harm, suicidal ideation, other)	Systemic and family therapy, CBT, mentalization‐based therapy	Counsellor/therapist, psychologist, psychiatrist
Law (2022)	N/A	United Kingdom	Empirical, journal article, narrative	Not reported	Multiple dx (trauma, general mental wellness)	Not reported	Counsellor, psychologist
Lee et al. (2022)	N/A	United States	Empirical, journal article, narrative	Youth, parents, caregivers, clinicians	Autism	Not reported	Not reported
Luebbe et al. (2018)	101–500	China	Empirical, journal article, quantitative	Youth, parents, caregivers, family members	Multiple dx (anxiety, general mental wellness)	Not reported	Not reported
Matalí et al. (2020)	101–500	Spain	Empirical, journal article, quantitative	Youth	Substance use	Not reported	Not reported
Maybery et al. (2013)	<100	United Kingdom	Empirical, journal article, quantitative	Children, youth, parents, caregivers	Multiple dx (families with substance use and/or mental health problems)	Not reported	Counsellor, psychologist, social worker
McGoron et al. (2014)	101–500	United States	Empirical, journal article, quantitative	Children, youth, parents, caregivers	ADHD	Not reported	Not reported
Parish (2020)	N/A	United States	Empirical, journal article, qualitative	Not reported	General mental wellness	Not reported	Psychologist
Pratt (2007)	N/A	Australia	Non‐empirical, narrative	Not reported	General mental wellness	Not reported	Psychologist
Penno et al. (2022)	<100	New Zealand	Empirical, journal article, qualitative	Youth	General mental wellness	CBT, IPT, mentalization‐based therapy, DBT, ACT	Counsellor, psychologist, psychiatrist
Rupani et al. (2014)	<100	United Kingdom	Empirical, journal article, mixed methods	Youth	Multiple dx	Not reported	Counsellor, psychologist, social worker
Salter et al. (2016)	<100	Australia	Empirical, journal article, quantitative	Children	Autism	Child‐centered play therapy	Counsellor
Selekman (2005)	N/A	United States	Empirical, book	Youth, parents, caregivers, clinicians	Multiple dx	Brief therapy, SFBT, strengths‐based approach	Psychologist
Selekman (2017)	N/A	United States	Empirical, book	Youth, parents, caregivers, clinicians	Multiple dx	Collaborative strengths‐based approach	Psychologist
Spinola et al. (2017)	101–500	United States	Empirical, journal article, quantitative	Youth	Substance use	Not reported	Not reported
Suarez‐Balcazar et al. (2022)	<100	United States	Empirical, journal article, qualitative (incl. case study)	Youth	Autism	Not reported	Not reported
Thompson and Sanchez (2012)	N/A	United Kingdom	Empirical, book	Youth, clinicians	Multiple dx	SFBT	Not reported
Walworth et al. (2009)	<100	United States	Empirical, journal article, quantitative	Youth	Autism	Music therapy	Counsellor
Kazdin and Weisz (2018)	N/A	United States	Empirical, book	Children, youth	Multiple dx	CBT	Psychologist
Wolpert et al. (2012)	501+	United Kingdom	Empirical, journal article, quantitative	Youth	Multiple dx (emotional disorder, conduct disorder, autism, ADHD, other)	Not reported	Not reported
Wolpert et al. (2015)	N/A	United Kingdom	Empirical, journal article, narrative	Not reported	Multiple dx	Not reported	Counsellor, psychologist, other mental health service providers
Wynne et al. (2016)	<100	Ireland	Empirical, journal article, quantitative	Youth	Multiple dx	Family therapy intervention	Psychologist, psychiatrist, social worker, nurse, occupational therapist, speech language pathologist
Zaitsoff et al. (2015)	<100	Canada	Empirical, journal article, qualitative	Youth	Eating disorder	Motivational interviewing	Counsellor

*Note*: Youth includes: adolescents, young adults age 10–25. Caregiver includes: parents or guardians. Type of provider refers to: counsellor/therapist as Master's level training or equivalent, psychologist refers to PhD level clinician.

Abbreviations: ABA, applied behaviour analysis; ACT, acceptance and commitment therapy; CBT, cognitive‐behavioural therapy; DBT, dialectical behaviour therapy; dx, diagnoses; IPT, interpersonal therapy; N/A, not applicable; SFBT, solution‐focused brief therapy.

The research team was comprised of individuals from diverse ethnic backgrounds, gender identities, lived experience with mental illness and neurodivergence, and varying levels of experience in youth mental health research. While our unique positionalities may have influenced the research, including the interpretation of findings, working collaboratively helped ensure the findings were deliberated on meaningfully. As such, our lived experiences also introduced situated knowledges (Haraway, 1998) into the research process that offered valuable insight, helped uncover gaps and elucidated opportunities for future work.

### Analysis

Data analysis was conducted collaboratively to mitigate potential biases while maintaining a shared comprehension of the data, the analytical process, and the overarching objectives. This approach aimed to establish “social reliability” (Sanders & Cuneo, 2010), a team‐based analytic practice designed to foster consensus and promote discursive agreement. Each publication was subjected to comprehensive coding through iterative cycles.

Thematic analysis was used to group practice principles. Grey literature findings were integrated with empirical research to provide a comprehensive, multi‐perspective synthesis. The final coding structure consisted of 16 main codes and 19 sub‐codes shown in Supporting Information S1: Table S1.

## RESULTS

Across the 53 publications included in the review, a total of 1279 text segments describing recommended approaches were extracted and coded, reflecting both specific clinical techniques (e.g., monitoring progress on goals) and broader practice principles (e.g., collaborative approaches to defining goals with youth). A median of two codes were applied to each segment, and the extracted recommendations were grouped into four domains of goal‐oriented practice. Table 1 provides a detailed breakdown of the characteristics and a summary of practice principles from the included publications (*k* = 53), consisting of 43 empirical journal articles, 1 non‐empirical publication, 1 dissertation, and 8 clinical guidebooks. Supporting Information S1: Figure S1 shows a heat map of themes and codes over time, including patterns and shifts in emphasis across the literature. The publications were heterogeneous in several aspects, including document type, geographic location, presenting problems or mental health diagnoses, as well as the types of providers and therapeutic approaches. Many publications included multiple comorbidities (*n* = 27, 50.94%) or discussed mental wellness more generally, and several publications focused on substance use health and addictions (*n* = 6, 11.32%) in particular.

The four main themes derived using the coding structure are shown in Table 2. Illustrative examples of each theme are provided in Supporting Information S1: Table S2.

**TABLE 2:** Overview of themes.

Themes and codes	Description
1. Semi‐structured goal formulation and progress monitoring	Focusing on the process of working with goals, the tools used for goal‐setting require thoughtful, context‐sensitive implementation, supported by appropriate measurement, monitoring, and practical strategies
Follow‐up and monitoring	Regular follow‐up at each or every few sessions should involve reviewing goals and evaluating progress, helping youth connect their actions to positive outcomes. Monitoring progress towards clearly defined goals highlights intermediate steps and can enhance patient engagement and self‐management. Discussing how to measure goals and consistently reviewing progress is vital for guiding treatment and ensuring goals remain relevant
Structures and strategies	Standard approaches in goal setting relies on consistent use of tools, strategies, and processes to support youth engagement and track progress over time. Goal setting commonly occurs with worksheets, progress charts, or digital platforms, and are integrated into session structure to facilitate regular measurement and discussion; tools including both informal discussion and idiographic measures like GAS or GBO, can be used to define and track goals data and changes over time. Useful goals are generally personalized, strengths‐based, attainable, and broken down into smaller, manageable steps
2. Collaborative youth‐ and family‐centered goal practices	Meaningful goal practices depend upon clinicians who are attuned to contextual, social, developmental, and cultural factors, and of engaging young people through shared decision‐making to ensure goals are collaboratively defined, meaningful and strengths‐based
Clinician values and qualities	Qualities such as patience, respect, and active listening, are essential for youth to take the lead in setting their goals and engaging with development of treatment plans. It is crucial that clinicians are aware of their own biases; ongoing training is essential to support clinicians in engaging in reflective practice. This practice signals genuine intention to care by encouraging dialogue and genuine interest in the youth's values and intentions.
Community context	Consideration of community or social context; connecting youth and families with community‐based resources can significantly help with goal attainment. Goals are deeply shaped by social and cultural environments, influenced by social learning and context. Consideration and discussion of social context is crucial as it impacts the perceived attainability and actual achievement of goals
Developmentally appropriate	Consideration of age, developmental capacity and readiness to engage in working with goals is crucial for individualizing treatment plans to a youth's specific needs and shaping outcomes. Further, youth receiving mental health treatment may have goals that differ significantly from their parents' goals; lack of clarity in developing goals may be prevalent with youth who may not have voluntarily sought treatment
Family informed	Accounting for caregiver/family impact on youth mental health treatment is essential for considering external factors affecting goal attainment; treatment planning supporting goal transfer outside of sessions. Ideally, goals should be developed collaboratively with caregivers/support‐persons, although clinicians often fail to explore youth and family dynamics and preferences regarding their involvement
Youth‐centered	Emphasis on agency, autonomy, and collaboration for youth to lead in defining their goals and preferences in how they work towards them. Supporting youth to be active participants in working with goals facilitates a sense of agency and inclusion. Clinical/treatment goals may differ from goals set by youth; young people often prioritize goals related to emotion regulation, self‐awareness, and relationships
3. Approaches for defining and tracking goals within therapeutic processes	The theoretical and clinical foundations that define the meaning of goals, their intended function in treatment, and how they work with therapeutic processes
Clinician‐set	Goals that are set by or perceived only by the clinician may not align with the youth's perspective and can limit therapeutic effort or focus on factors that are less important to the youth. Assessing progress towards goals often correlates with clinician‐reported functioning improvement, therefore, standardized tools may not fully capture the youth's personal experience of change
Conceptualized	Theoretical and conceptual framing of goals and progress including motivation, engagement, and change. Visualizing interventions and progress, through methods like rating scales or vision boards, can provide representation of underlying therapeutic constructs and practical aid for creating goals. Systematic documentation and review of goals helps to make abstract goals more concrete and support shared conceptual framework between the youth and clinician
Domain‐specific	Goals can be categorized into various taxonomies or domains such as interpersonal, emotional, or personal growth, each reflecting different concepts that are meaningful to the youth. This categorization can be helpful for youth and clinicians to understand the range and depth of goals and adjust approaches
Flexible	Goals should be thought of as flexible and modifiable rather than fixed endpoints, allowing for revision as new challenges arise or context shifts. It's important to check‐in with youth to evaluate if the initial goals are still relevant and aligned with their current needs
Negotiated	Working with goals is a collaborative process where clinicians work with clients and customize interventions/approaches to their unique needs and values. Negotiating goals ensures they are individualized and jointly agreed upon by all relevant parties, including youth and families as relevant. If initial goals are unrealistic or unspecific, discussion is necessary to establish more attainable options or steps
Reflection	Goal attainment is intrinsically linked to self‐regulation, which includes the process of setting goals and reflecting on progress/challenges. Both clients and clinicians benefit from reflecting on progress, whether through recording small steps or analyzing data. It is important for clinicians and youth to collectively recognize and celebrate progress along the way, rather than only focusing on the final goal achievement
Tailored	Tailoring strategies to match the unique needs, priorities, values, and mental health strengths/challenges of each youth. Confidence in their ability to change and agreement on goals are crucial for engagement and making treatment gains. The relationship between factors like motivation and goal setting is dynamic; symptom severity and goals often fluctuate over time
Therapeutic approach	Offering youth choice in therapeutic processes and approaches can improve motivation and lead to better outcomes. While goal‐oriented practice is core to most therapies, modalities differ in when and how goals are used. Consideration of congruence between youth's individual goals and therapeutic approach and tools is important for effective intervention
4. Cultural safety and EDI in goal practices	Meaning of cultural safety and equity is often assumed and not clearly articulated at both individual‐ and systems‐levels; requires that clinicians recognize potential power imbalances and assess the limitations/biases of routine therapeutic approaches Cultural background and identity of both youth and clinicians significantly influence the content of goals, understandings of mental health and mental health treatment, and readiness for change. Concepts often associated with goal‐setting, such as independence, productivity, and empowerment, are Western values and may not universally resonate with youth from diverse backgrounds

Abbreviation: EDI: equity, diversity, and inclusivity.

### Summary of practice principles themes

#### Semi‐structured goal formulation and progress monitoring

Structures and strategies for working with goals was commonly identified, with 49 publications (92.45%) addressing tools, measures, and timing. Various measures were described or recommended in 48 publications (90.57%); 35 (66.04%) recommended measurable goals, 31 (58.49%) described specific tools or frameworks (i.e., patient‐reported outcome measures), and 15 (28.30%) described non‐standardized or creative approaches. Consideration of timing and follow‐up of goals was recommended in 31 publications (58.49%) e.g., follow‐up at each session (Thompson & Sanchez, 2012; Cook‐Cottone et al., 2019; Wolpert et al., 2015), weekly discussion of goals (Zaitsoff et al., 2015; Kazdin & Weisz, 2018) at the beginning and end of an intervention spanning several weeks or months (Rupani et al., 2014; Spinola et al., 2017; Wynne et al., 2016)), or reviewing goals again at an unspecified time in the future (Cooper & Law, 2018, Jacob et al., 2022; Edbrooke‐Childs et al., 2015)), and 22 (41.51%) endorsed refining goals to be more attainable. Incorporating psychoeducation was described in 21 publications (39.62%), multi‐stage approaches were recommended in 15 publications (28.30%), and using SMART goals was described in 15 publications (28.30%). Limiting the number of goals to between one and three was recommended in 11 publications (20.75%), and four publications (7.55%) identified barriers to working with goals with youth in mental health treatment (e.g., navigating unobtainable goals, perceived inflexibility of standardized measures of client outcomes when working with subjective goals rather than behaviour‐focused goals (Cooper & Law, 2018); external social or structural factors that limit goals (Bhattacharya, 2021); building trust with clients who are in a state of epistemic hypervigilance (Law, 2022); avoidance of goals talk or anxiety surrounding future‐oriented goals which in turn can negatively impact therapeutic alliance (Penno et al., 2022)).

#### Collaborative youth‐ and family‐centered goal practices

Approaches to working with goals that prioritize youth and family perspectives was also identified as a theme in 49 publications (92.45%). Youth‐centered approaches to working with goals were recommended in 36 publications (67.92%), emphasizing autonomy, collaboration, and alignment of goals with the young person's values. In contrast, family‐centered approaches were recommended in 27 publications (50.94%),including caregiver involvement in both goal development and attainment. Clinician values and qualities were described in 24 publications (45.28%) including 14 (26.42%) publications that advocated for fostering a safe space and supporting the therapeutic alliance, 13 (24.53%) that recommended specific clinician behaviours that support goal setting (e.g., when using a tool like the GBO, providing feedback or debriefing with the youth can provide added perceived value of these tools (Duong et al., 2016); asking caregivers and youth about their goals and integrating elements of both into a plan (McGoron et al., 2014); formulating youth's personal goals to align with short and long‐term goals that complement objectives for clinical outcomes (Hurlburt et al., 2010)), and 10 (18.87%) that endorsed self‐reflection or professional development. Addressing the role of broader social contexts to support the process of working with goals was recommended by 20 publications (37.74%). Ensuring developmental appropriateness of goals was recommended by 8 publications (15.09%).

#### Approaches for defining and tracking goals within therapeutic processes

Conceptualizing goals across treatment was identified as a theme in 46 publications (86.79%). The process of creating goals was described in 31 publications (58.49%), with variable approaches: 10 (18.87%) proposed visual or creative approaches, nine (16.98%) recommended writing or journaling, nine (16.98%) supported conversational approaches, and four (7.55%) endorsed numerical methods with scores or rating systems. Specific therapeutic approaches (e.g., CBT, dialectical behaviour therapy) were described in 25 publications (47.17%). Flexible approaches were emphasized by 16 publications (30.19%). Collaboration in refining goals with youth and caregivers was recommended in 14 publications (26.42%), whereas clinician‐led goals were recommended in 13 publications (24.53%). Additional theoretical codes included reflection on goal progress recommended in 15 publications (28.30%), 21 (39.62%) proposed framing goals within taxonomies, and 12 (22.64%) described goals and tailored to specific diagnoses.

#### Cultural safety and EDI in goals practice principles literature

Cultural safety in relation to working with goals was discussed in 17 publications (32.08%), and EDI‐related content was identified in 18 publications (34.00%). Although there was an increase in references to EDI principles over time, particularly from 2018 onwards, these segments were often brief and lacked depth or meaningful description (e.g., “Content of goals are dependent on social and cultural norms” (Rupani et al., 2014); “Clients come to counseling with a variety of expectations, goals, and values stemming from their individual experiences and diverse backgrounds.” (Dickens, 2020)).

## DISCUSSION

This review aimed to extract and synthesize recommended approaches for working with goals with youth aged 10–25, and to examine how equity, diversity, and inclusion (EDI) considerations are embedded within those practices. The findings suggest that goal‐oriented approaches are tied to both broader therapeutic competencies—such as strengthening therapeutic alliance and supporting shared decision‐making—and to specific techniques and strategies that characterize effective goal related practice (e.g., setting goals early in treatment, combining standardized and idiographic measures). This review also found that, while references to EDI have increased in recent years in the goal‐oriented practice literature, these discussions were generally limited in depth, making it challenging to assess how equity‐informed principles may be specifically operationalized within clinical practice. While many studies advocate for goal‐setting as a mechanism to enhance engagement and outcomes, few offer structured approaches that explicitly address cultural safety, identity, or systemic barriers to goal attainment.

### Formulating and monitoring goals

Routine progress monitoring has become increasingly common in youth mental health and addiction services, often supported by digital platforms and feedback tools (Cairns et al., 2015; Edbrooke‐Childs et al., 2015; Hurlburt et al., 2010; Jacob et al., 2017). Studies in this review used visual and technological aids to help youth track goals over time, reflecting a growing emphasis on transparency and shared accountability. Personalized, idiographic measures such as the GBO tool—one of the few PROMs designed specifically for youth—can help center young people's voices in treatment. However, their effective use depends on clinicians' ability to navigate contextual, social, developmental, and cultural factors (Cairns et al., 2019; Cooper & Law, 2018; Jacob et al., 2017). Few studies explicitly address how to prioritize youth‐defined goals or gave examples of how to support youth agency within these practice (Alderson et al., 2019; Jacob et al., 2017; Penno et al., 2022), underscoring a gap in guidance around meaningful goal negotiation and renegotiation. Given that youth report mixed experiences with goal‐setting (Duncan et al., 2025) the importance of distilling what these practices look and feel like in situ are vitally important to ensure quality care.

Youth‐ and client‐centered practice and strengths‐based approaches to goal‐formulation and monitoring have become more prominent recommendations from 2017 onwards, emphasizing working with goals as a way to foster a sense of agency and motivation (e.g., Cooper & Law, 2018; Jacob et al., 2017; Kanter et al., 2021; Selekman, 2017). The literature increasingly frames goal work as a dynamic process—one that involves continuous reflection, discussion, and adjustment as youth needs evolve (Alderson et al., 2019; Cairns et al., 2019; Jacob et al., 2022). While some studies recommend involving caregivers or family members (Dickens, 2020; Fiks et al., 2013; Hurlburt et al., 2010), there is limited differentiation in how this involvement should vary based on age, capacity, or youth preferences (Hanson & Lynch, 2013; McGoron et al., 2014). Moreover, the clinician's role in navigating conflicting goals between youth and caregivers is rarely explored, despite its relevance to real‐world practice (Day et al., 2017; Parish, 2020; Suarez‐Balcazar et al., 2022).

### Equity, diversity and inclusivity

This review underscores a persistent disconnect between the growing emphasis on goal‐oriented care in youth mental health and the limited guidance on how to implement these practices equitably and inclusively. Much of the available literature addressing EDI considerations highlighted that standard approaches or frameworks for working with goals may not translate across cultural contexts. Several studies focused on disparities in service access and engagement among racialized youth (Kazdin & Weisz, 2018), as well as Western‐centric assumptions embedded within constructs related to goals such as empowerment and self‐direction (Chandrasekhar et al., 2023; Suarez‐Balcazar et al., 2022). However, these discussions generally provided limited guidance on how clinical practice could be adapted in response. Similarly, while tools like the GBO measure offer promise in centering youth voices, few studies in this review addressed how youth identities‐such as race, gender, disability, or cultural background ‐ intersect with goal formulation. Where specific practices or approaches were described, they focused primarily on tailoring broader therapeutic practices to families' cultural needs and perspectives (Francis et al., 2021; Hanson & Lynch, 2013). One publication by Pratt (2007), stood out for its early and more critical perspective on cultural safety and EDI in working with goals in mental health treatment, drawing on reflections from the author, an Indigenous Australian counsellor working with Aboriginal and Torres Strait Islander people. Pratt emphasized the importance of patient‐defined safety, cultural relevance, and practitioner reflexivity in goal‐setting, offering deeper insights into how structural forces influence therapeutic engagement. To move forward, expanded frameworks are needed that are not only evidence‐based but also values‐driven, structurally responsive, and informed by more diverse youth and clinician perspectives (Duncan et al., 2025; Guinaudie et al., 2020; Henderson et al., 2022; Law, 2019; Watson et al., 2023).

### Limitations

Currently available source literature may reflect idealized research perspectives rather than the practical realities of clinicians. Many of the recommended practices were presented aspirationally, with few providing clear evidence of their feasibility or direct impact in real‐world contexts; explicit guidance on how clinicians should operationalize goal‐oriented practices was often limited or inconsistently described, which contributed to variability in the specificity of recommendations across sources (Jacob et al., 2017, 2023; Penno et al., 2022). However, recent publications from Chiodo et al. (2025), Duncan et al. (2025), and Mok et al. (2024) offer promising evidence that begin to address this gap. Second, we do not know whether additional sources, or those in other languages would have yielded additional principles and/or practices. An additional consideration relates to the organization of youth mental health and addictions services, with some systems integrating these supports within shared models of care while others continue to provide these through separate pathways. As the literature included in this review reflected both integrated (Cairns et al., 2019; Cooper & Law, 2018) and distinct service contexts (Alderson et al., 2019; Kaminer et al., 2018; Spinola et al., 2017) future work should examine whether and how recommendations for working with goals may differ across these service delivery models with youth populations (Magill et al., 2022). Lastly, the quality of the included publications was not formally assessed which restricts the ability to evaluate the methodological rigor and reliability of the evidence informing goal‐oriented practices.

## CONCLUSION

Practice principles for working with goals in youth mental health are essential for translating the growing emphasis on these approaches into consistent, equitable, and effective practice. Despite widespread recognition of the benefits of working with goals, current guidance is fragmented and often lacks the procedural clarity and structural responsiveness needed to support clinicians in engaging diverse youth populations specifically. This review highlights the absence of equity‐informed frameworks that connect individual‐level goal work with broader contextual factors such as cultural identity, systemic barriers, and power dynamics. Future guidelines must be both evidence‐based and values‐driven ‐ supporting clinicians and youth to work collaboratively with goals in ways that are likely to yield positive outcomes but that are inclusive and attuned to the evolving priorities of youth.

## AUTHOR CONTRIBUTIONS

**Madison Nugent**: Conceptualization; methodology; formal analysis; investigation; data curation; writing—original draft; visualization. **Drew Burchell**: Investigation; formal analysis; writing—review and editing. **Jill Chorney**: Conceptualization; writing—review and editing. **Sharon Clark**: Conceptualization; writing—review and editing. **Mick Cooper**: Conceptualization; writing—review and editing. **Debbie Emberly**: Conceptualization; writing—review and editing. **Danielle Impey:** Conceptualization; writing—review and editing. **Jenna Jacob**: Conceptualization; methodology; investigation; writing—review and editing; funding acquisition. **Anam Khan**: Data curation; visualization; writing—review and editing. **Jennifer Lane**: Conceptualization; validation; writing—review and editing. **Duncan Law**: Conceptualization; validation; writing—review and editing. **Rebecca Pillai Riddell**: Validation; writing—review and editing. **Lori Wozney**: Conceptualization; project administration; supervision; methodology; formal analysis; writing—original draft; funding acquisition.

## CONFLICT OF INTEREST STATEMENT

The authors declare no conflicts of interest.

## ETHICAL CONSIDERATIONS

Review study, research ethics review not applicable.

## Supporting information

Supporting Information S1

## Data Availability

The data that support the findings of this study are available within the article and its supplementary materials.
