# Supplementary material for: Working with goals in youth mental health and substance use service: A review of current practice guidelines and recommendations
Source: JCPP Adv. 2026 Oct 7:e70173. Online ahead of print. doi: 10.1002/jcv2.70173 (PMC13644939; doi:10.1002/jcv2.70173)
Supplement: Supplementary file 1 [file JCV2-9999-e70173-s001.docx]

**Working with Goals in Youth Mental Health and Substance Use Service: A Review of Current Practice Guidelines and Recommendations**

**Supporting Information**

**Table S1.** *Coding Structure*

| Themes and Codes | | | Definition |
| --- | --- | --- | --- |
| 1. Semi-Structured Goal Formulation and Progress Monitoring | | | |
|  | Follow-up and Monitoring | | Discussion about timing of goal-setting; having a structured plan for checking in and following up on progress of goals. |
|  | Structures and Strategies | | Specific approaches, tools, strategies, and processes for working with goals. |
|  |  | Measurable | Goals that have a clearly defined desired outcome. |
|  |  | Standardized | Use of specific or validated tool or process for scoring, grading, monitoring progress toward desired outcome. |
|  |  | Non-Standardized | No specific or systematic tool for monitoring goals and/or not using formal “goal” language. |
|  |  | Realistic | Achievable goals taking into consideration individual client strengths and limitations. |
|  |  | Barriers to Working with Goals | Personal and contextual factors that limit ability to work with goals. |
|  |  | Stepwise | In setting and working towards goals, best practice is discussed as breaking down goals into manageable steps or stages (e.g., interim goals, multiple steps, smaller goals leading into more significant goals). |
|  |  | SMART Goals | Framework for setting goals that are specific, measurable, achievable, relevant, and time-bound. |
|  |  | Limited | Focus on setting a smaller/set number of goals (e.g., three or less). |
|  |  | Scaffolded | Education about goal setting provided (e.g., detailed written information on the purpose and process of goal setting, instruction on how use goals measures, selecting realistic goals). |
| 2. Collaborative Youth- and Family-Centered Goal Practices | | | |
|  | Clinician values and qualities | | Qualities, traits, or characteristics described as important for the therapeutic relationship and for the use of goals in therapy with youth. |
|  |  | Clinician Traits | Specific qualities or behaviours that support working with goals (e.g., empathy, listening, respect, patience, motivational, responsive to feedback). |
|  |  | Safe and Power Aware | Focus on how clinicians position themselves within the therapeutic relationship to empower youth or create a safe environment that addresses power dynamics. |
|  |  | Continually Improved | Goal-oriented practice skills of the clinician described as an evolving process that requires learning about diverse youth and their experiences. |
|  | Community Context | | Goals developed and monitored centering aspects of community, social, and environmental context. |
|  | Developmentally Appropriate | | Goals and goal-setting process being adjusted or reflective of developmental stage of youth. |
|  | Family Informed | | Family members consulted on youth goals or family members setting their own goals. |
|  | Youth-Centered | | Focus on goals relevant and important to the youth to promote ownership and autonomy. |
| 3. Approaches for Defining and Tracking Goals Within Therapeutic Processes | | | |
|  | Clinician-Set | | Goals developed based on therapist assessment of what youth needs without youth being involved in the goal setting process. |
|  | Conceptualized | | Approaches for creating the goal or capturing progress. |
|  |  | Visual | Whiteboard drawings, graphs, art-based, drama. |
|  |  | Numeric | Using numbers to capture goal intensity, goal progress, goal difficulty, goal priority. |
|  |  | Written | Focus on having youth write down, often in their own words, their own goals. |
|  |  | Verbal | Focus on having youth articulate their goals out loud/verbally. |
|  | Domain-Specific | | Goals set within defined impairment or functional areas or taxonomies. |
|  | Flexible | | The ability to modify goals with changing youth priorities/needs. |
|  | Negotiated | | Ability to navigate and (re)negotiate goals when there is not initial agreement between youth and others involved. |
|  | Reflection | | Clinician acknowledges changes client has made, affirms accomplishment of goals. |
|  | Tailored | | Goals used in particular ways for specific mental health or substance use challenges or disorders. |
|  | Therapeutic Approach | | A specified therapeutic approach is recommended for working with goals with youth. |
| 4. Cultural Safety and EDI in Goal Practices | | | Focus on adapting approaches, centering aspects of identity and social determinants of health in how goals are set or monitored. |

*Note.* EDI, equity, diversity, and inclusivity.

**Figure S1.** *Temporal heat map of themes across publications, 2005-2023*

*Note.* Each cell reflects number of publications assigned a specific code in a given year, grouped into the four main themes. Darker shades indicate a higher number of publications coded, highlighting shifts in focus and coding frequency over time. n, frequency; EDI, equity, diversity, and inclusivity.

**Table S2.** *Illustrative examples of themes for working with goals with youth in community mental health and addictions treatment*

| **Themes and Codes** | | **Examples** |
| --- | --- | --- |
| **1. Semi-Structured Goal Formulation and Progress Monitoring** | | |
|  | Follow-up and Monitoring | "Review goals, evaluate progress and make future plans; Consolidate personally meaningful change descriptions and/or "new'' identity; Develop connection between participants' actions and positive outcomes; Acknowledging and complimenting goal accomplishments; Celebrating changes: Ownership of goal accomplishments" Thompson & Sanchez (2012) |
|  |  | "discuss how the goal can be measured together with the client when the goal is set. The might be a numerical or frequency measurement, might relate to keeping a diary of thoughts and feelings, or might refer to being able to do or not do things on a number of occasions" Cooper & Law (2018) |
|  |  | "Once your goals are set, address them at each session, assessing and monitoring progress. This can help guide you as you will have a documented sense of what is working and what is not and when you might need to change course, increase or decrease intensity and/or frequency, or secure additional support or a higher level of intervention" Cooke-Cottone et al. (2019) |
|  | Structures and Strategies | "‘In coming to this service, what are some of the problems you want help with or goals you want to get to?’ The GBO tool records three main goals, but it also gives users the option to note any additional goals they might have." Rupani et al. (2014) |
|  |  | "GAS graphing (see Figure 1) provided a useful visual reminder to both therapist and parent of the targeted goal and child progress" Salter et al. (2016) |
|  |  | "‘SMART’ acronym is used as the basis for goal setting (specific, measurable, achievable, realistic, time-limited) […] Goal-setting may be undertaken with clients in a relatively informal and 'low-key' way. For instance, it may simply involve asking clients about the things they want in therapy, and keeping a mental note of this as the therapeutic work progresses." Cooper & Law (2018) |
|  |  | "Youth are invited to set, usually, up to three goals about what they want to be different in their lives as a consequence of engaging in therapy, and rate progress toward these goals on a simple, 11-point scale." Law (2022) |
| **2. Collaborative Youth- and Family-Centered Goal Practices** | | |
|  | Clinician Values and Qualities | “honor their long problem-saturate stories, to be a better listener, to be patient, to be respectful of their pain and frustration, to be in awe of their persistence and resiliency, and to empower them to take the lead voice with goal setting and treatment planning.” Selekman (2005) |
|  |  | “goal-oriented treatment; focusing on the positive; being nonblaming; learning as self-focused and noninstructional; emphasis on fostering new, beneficial thinking and behaviors; helping participants to focus on small, attainable changes that provide indicators of progress; and emphasizing solutions coming from within.” Thompson & Sanchez (2012) |
|  |  | “It is vital to our work as counselors to continuously check in on our potential biases, beliefs, and attitudes, and evaluate levels of privilege and marginalization within the counseling relationship.” Dickens (2020) |
|  |  | “More time and careful consideration of appropriate language may be needed, along with training and support to clinicians to facilitate goals that are genuinely privileging young people's own hopes for their future. The 'task' of setting genuinely collaborative agreed goals that are safe, and fit with the context and remit of the service, is more challenging.” Jacob et al. (2022) |
|  | Community Context | “emphasis on linking youth and families with community activities and resources was intricately associated with goal attainment […] inclusion of such informal helpers (e.g., relatives, friends, neighbors, associates from church or other faith communities) in the wrap-around team process of service planning and delivery has long been considered a mainstay of this and other community-based approaches” Cox et al. (2010) |
|  |  | “Goals form an essential part of intentional self development and are reflected through an individual's plans, projects and course of action in which one invests time and effort. The implementation of these goals depend on numerous factors like, the way in which the goal is interpreted, means necessary for attaining the goal and whether the means to achieve the goal are available at personal and social level, as well as the perceived barriers which may hamper the attainment of the goals” Bhattacharya et al. (2013) |
|  |  | “Social factors can determine goals, and goal-related processes, in many ways. First, the kinds of goals that people adopt- right up to their highest order goals - are likely to be learnt from their social and cultural environments. Indeed, 'there is research to suggest that goal pursuit is automatically triggered when goals are inferred from the behavior of others', a process known as 'goal contagion'.” Cooper & Law (2018) |
|  | Developmentally Appropriate | “The assessment results of a child's need area(s) formulates the intervention plan and techniques used, and therefore shapes the possible outcomes of the child.” Walworth et al. (2009) |
|  |  | “Children who are therapy clients also tell us that they have a specific difficulty which does not normally relate to adults. This is when their parents' goals and their own are very different. […] one of the challenges you may face as a therapist is a client simply not knowing what goals to set. Many clients will not have walked through the door with pre-set goals. This may be particularly so for children and young people who may not have been involved in deciding they want or need therapy. It is important that, however old the client is, they are told that it is 'ok' not to have clear goals at the start” Cooper & Law (2018) |
|  | Family Informed | “the therapist must appreciate the significance of these in reference to the person belonging to a family, community and culture” Pratt (2007) |
|  |  | "With parent involvement in treatment planning and intervention sessions, parents play an integral role in the transference of treatment goals outside of the therapy session. Parent training can empower parents with positive interaction tools outside of the therapy session” Walworth et al. (2009)" |
|  |  | “family culture and perspectives regarding mental health are also an important consideration to take into account, as these perspectives influence the degree to which families discuss mental health and how they approach mental wellness for their child” Francis et al. (2021) |
|  |  | "clinicians sometimes fail to explore families' preferences and goals regarding treatment” Fiks et al. (2013) |
|  |  | "There also appears to be some tendency for counsellors to over-emphasise the centrality of family and relationship concerns. In general, the pattern seems to be that young people are more likely to describe their goals in terms of changing how they feel; while counsellors are more likely to describe the presenting and developing problems in terms of contextual issues, and particularly relationships with their family.” Rupani et al. (2014) |
|  | Youth-centered | ""what are your best hopes out of our work together?" He adds, "To the client, come several important messages: the therapist's belief that the client's agenda is paramount, that the client has agreed to the meeting for a purpose and, perhaps most important of all, the therapist has put him or herself entirely in the hands of the client since no one else in the entire universe can answer this question"" Selekman (2017) |
|  |  | "youth, see themselves as an active agent in their own development, set personal growth goals, experience a sense of cognitive engagement in such goals and attempt to work towards realizing such goals" Bhattacharya et al. (2013) |
|  |  | "The 'process' of having goal-focused conversations is a vital part of any intervention assessment, it facilitates a sense of agency and inclusion with young people by involving them in decisions about their care, and is the basis for informed consent. [...] young people then become 'participants' and not just 'patients'. It is this added agency that may explain emerging evidence suggesting positive links between collaborative goal setting and good mental health outcomes" Jacob (2022) |
| **3. Approaches for Defining and Tracking Goals Within Therapeutic Processes** | | |
|  | Clinician-Set | “If a therapist perceives that a goal has been pursued, it may limit the therapist’s further efforts to pursue the same objective […] Therapists might also attempt to build upon goals that they perceive to have been a focus in previous sessions, whereas a client might not have registered it as similarly significant” Hurlburt et al. (2010) |
|  |  | “change in progress toward goals was uniquely significantly associated with change in clinician-reported functioning, while change in psychosocial difficulties and impact on daily life were not, and clinicians reported higher levels of improvement in functioning for children and young people who made more progress toward achieving goals.” Edbrooke‐Childs et al. (2015) |
|  |  | “as normed, standardized tools track change using professionally defined items (i.e., clinically determined), they may not capture the issues most relevant to the client and therefore may underestimate the magnitude of change experienced by the client themselves” Jacob et al. (2023) |
|  | Conceptualized | “At each review, families rated each goal as achieved (3), good progress (2), some progress (1) or not achieved (0). This enabled an assessment of progress for families and also allowed a calculation of change for each goal for each family member.” Maybery et al. (2013) |
|  |  | “Vision boards can be used to inspire and help clients to focus on their goals and to empower them to create the life they imagine. Aligned with solution-focused therapy and imagery, clients become responsible for guiding their outcome goals in counseling, which helps clients illuminate for themselves what is important to them.” Burton & Lent (2016) |
|  |  | “Both teens and clinicians found value in reflecting on the data summaries and graphs and discussing it together. Clinicians wanted to use the data summary and visualizations to identify activities and contexts that triggered negative emotions and the intensity of the emotion, identify positively influencing activities, and plan future goals and recommendations with teen clients” Bhattacharya (2021) |
|  | Domain-Specific | “measure the perceived importance of four life goal categories (Relations, Generativity, Religion and Achievements)” Gabrielsen et al. (2012) |
|  |  | “Goals nominated by participants are categorised into corresponding goal categories including: educational, career, interpersonal, delinquency, freedom-autonomy, self-presentation, physical, and reputation. The participants rank their goals in order of importance.” Carroll et al. (2013) |
|  |  | “Young people report that goals help to prioritize the target of treatment, although they may be concerned that goal achievement will result in treatment termination. Young people have been shown to set three broad types of goals in CAMHS: relationship/interpersonal, coping with specific problems and symptoms, and personal growth and functioning” Edbrooke‐Childs et al. (2015) |
|  |  | “specific goals allow the counselor and the client to confidently gauge the success of the counseling” Burton & Lent (2016) |
|  | Flexible | "revised in light of new challenges or goal completion" Maybery et al. (2013) |
|  |  | "not treating the client's goals as fixed and definitive endpoints for therapy - which must be achieved at any costs - but as fluid, changeable, and, inevitably, approximate indicators of the client's preferred 'direction of travel' [...] 'Is there anything that we could do differently in this session which might help things move forward? Is there anything I could do that might make things more helpful?' This could also include questions about the goals themselves: 'Do the goals we set at the beginning of the intervention still feel the right ones that you want to work towards?'" Cooper & Law (2018) |
|  |  | "Goals may change over the course of care, and it is important to balance this with the complexity of tracking progress over time. Clinicians might instead work flexibly with patients and deviate from goals as required, perhaps treating them as guidance topics to remain on track. However, in order to be effective, goals must be individually tailored and collaboratively used." Jacob et al. (2022) |
|  | Negotiated | "In optimal service delivery, practitioners use various strategies to enable/empower clients and customize interventions, resulting in individualized therapy goals and treatment plans developed jointly with clients. […] Customizing strategies involve matching the intervention to unique goals and needs of clients and ensuring the appropriateness of service" King (2009) |
|  |  | "Clinicians, children, and parents - independently or, ideally, jointly - agree upon their goals and rate progress toward achieving these over the course of treatment" Edbrooke-Childs et al. (2015) |
|  |  | "What clients tell us is important is the discussion and deliberation to understand together why this may be unobtainable and to work together to agree what may be a more realistic goal" Cooper & Law (2018) |
|  | Reflection | "Goal attainment reflects the ultimate purpose of self regulation. Goals are involved across different phases of self regulation. 1. Forethought (setting a goal and deciding on goal strategies). 2. Performance control (employing goal-directed actions and monitoring performance). 3. Self-reflection (evaluating one's goal progress and adjusting strategies to ensure success)." Bhattacharya et al. (2013) |
|  |  | "concentrate on self-reflection, incorporating identifying and working collaboratively towards goals" Jacob et al. (2017) |
|  |  | "Both teens and clinicians found value in reflecting on the data summaries and graphs and discussing it together. Clinicians wanted to use the data summary and visualizations to identify activities and contexts that triggered negative emotions and the intensity of the emotion, identify positively influencing activities, and plan future goals and recommendations with teen clients" Bhattacharya (2021) |
|  |  | "be cognisant that goals belong to the young person, are important to them, and therefore any progress along the journey can be celebrated, not just the ultimate achievement of goals." Jacob et al. (2022) |
|  | Tailored | "Customizing strategies involve matching the intervention to unique goals and needs of clients and ensuring the appropriateness of service. Therapists use customizing strategies to focus and pace the intervention; plan an intervention experience that fits the needs, concerns, priorities, values, and worldviews of clients; and ensure that the intervention process is appropriate and feasible for a particular family" King et al. (2009) |
|  |  | "treatment priority goals" Walworth et al. (2009) |
|  | Therapeutic Approach | "when clients are given a choice regarding treatment goals and the type of treatment they want to receive, they will be more motivated and have more favorable treatment outcomes" Selekman (2005) |
|  |  | "By taking a strengths-based approach, in which attention is focused on client abilities, resources, and opportunities, practitioners are able to see possibilities and establish joint visions with families" King et al. (2009) |
|  |  | "young people’s therapeutic goals may be relatively independent of the particular kind of therapy on offer or on the therapeutic context." Rupani et al. (2014) |
|  |  | "Differences are found across these approaches in the methods used to negotiate goals, and the extent that these are made explicit and embedded in the work. Greater similarities are found in the use of goals in CBT and IPT, compared with the less directive modalities of humanistic, psychodynamic, and systemic psychotherapies. Differences are also found within modalities (e.g. psychoanalytic psychotherapy for adults versus psychoanalytic child psychotherapy)." Cooper & Law (2018) |
| **4. Cultural Safety and EDI in Goal Practices** | | |
|  |  | “The therapist must be cognisant of their role in not only promoting power imbalances between therapist and the Indigenous person, but also the wider social network that is Indigenous culture and identity as represented by personal, familial and historical stories.” Pratt (2007) |
|  |  | “important for me to consider how clients’ gender, race, culture, ability, sexual and affectional orientations, religion, age, and ethnicity factor into their readiness and openness for change.” Dickens (2020) |
|  |  | “Concepts associated with goal-setting such as self-control, independence, and empowerment represent Western values and are often hard to understand by working-class populations who have a history of marginalization and/or non-Western cultures.” Suarez-Balcazar et al. (2022) |

*Note.* EDI, equity, diversity, and inclusivity.
